# Peer Review of “In-hospital Mortality and the Predictive Ability of the Modified Early Warning Score in Ghana: Single-Center, Retrospective Study”

**DOI:** 10.2196/30785

**Published:** 2021-07-12

**Authors:** John Mogaka

**Affiliations:** 1 Department of Public Health Medicine University of KwaZulu Natal Durban South Africa; 2 Research Xcellas Durban South Africa

**Keywords:** modified early warning score, MEWS, AVPU scale, Korle-Bu Teaching Hospital, KBTH, Ghana, critical care, vital signs, global health

*This is a peer-review report submitted for the paper “In-hospital Mortality and the Predictive Ability of the Modified Early Warning Score in Ghana: Single-Center, Retrospective Study.”*

## Round 1

### General Comments

This study [1] is about a measure of illness severity that can potentially promote the early detection of clinical deterioration in critically ill patients. More specifically, the study investigated in-hospital mortality and the predictive ability of a modified early warning score (MEWS) in Ghana. By employing receiver operating characteristic (ROC) curves and other statistical techniques, the authors validated a limited MEWS (LMEWS). Finding a promising measure of instances of clinical deterioration is valuable for the timely and proper management of acute deterioration events in clinical settings. Though this paper seems to have made contributions to the medical field, there are some issues worthy of consideration.

### Specific Comments

#### Major Comments

1. One of the main concerns about this study is that the sample size is relatively small (N=112) for a national referral hospital in Ghana. Authors should provide more evidence on whether the sample and size were representative of the target population. Relatedly, since the authors state that they recruited practically all medical inpatients hospitalized for a period of more than 2 years (January 2017 to March 2019), it would be good to provide the total recorded number of in-hospital patients for that period.
2. In making the case for the validity of LMEWS, the authors have relied heavily on the afferent arm of clinical deterioration in critically ill patients, while not accounting for the efferent arm of medical response. The afferent arm identifies patients at risk of clinical deterioration and activates the efferent arm if necessary. The efferent arm examines the patients and intervenes in the treatment. The functioning of the efferent arm in the study settings ought to have been discussed in drawing up the conclusion and recommendation of the LMEWS.

## References

[1] Abbey EJ, Mammen JSR, Soghoian SE, Cadorette M, Ariyo P (2021). In-hospital Mortality and the Predictive Ability of the Modified Early Warning Score in Ghana: Single-Center, Retrospective Study. JMIRx Med.

